# Finite Element Analysis of Stress Distribution in Healthy and Restored Mandibular Molars with Zirconia and Lithium Disilicate Crowns Under Vertical and Oblique Loading

**DOI:** 10.3390/jfb17080404

**Published:** 2026-08-14

**Authors:** Rosa Alicia Hernández-Vázquez, Rodrigo Arturo Marquet-Rivera, Octavio Alejandro Mastache-Miranda, Karina Gabriela Madrigal-Carrillo, Rosa Adriana Rivera-Díaz

**Affiliations:** 1 División de Mecatrónica, Universidad Politécnica del Valle de México, Avenida Mexiquense sin Número Esquina Avenida Universidad Politécnica, Colonia Villa Esmeralda, Tultitlán 54910, Mexico; alicia.hernandez@upvm.edu.mx; 2 Posgrado, Facultad de Odontología, Universidad Latinoamericana Campus Norte, Cumbres de Acultzingo 3, Fraccionamiento Los Pirules, Tlalnepantla de Baz 54040, Mexico; 3 Escuela Superior de Comercio y Administración Unidad Tepepan, Instituto Politécnico Nacional, Anillo Periférico Sur Manuel Gómez Morín 4863, Colonia Ampliación Tepepan, Alcaldía Coyoacán, Ciudad de México 16020, Mexico; 4 Escuela Superior de Ingeniería Mecánica y Eléctrica Unidad Culhuacán, Instituto Politécnico Nacional, Avenida Santa Ana 1000, San Francisco Culhuacán, Colonia Culhuacán CTM V, Alcaldía Coyoacán, Ciudad de México 04440, Mexico; 5 Unidad Profesional Interdisciplinaria de Energía y Movilidad, Instituto Politécnico Nacional, Avenida Wilfrido Massieu sin Número, Unidad Profesional Adolfo López Mateos, Nueva Industrial Vallejo, Gustavo A. Madero, Ciudad de México 07738, Mexico; 6 MARMAS Soluciones en Ingeniería e Investigación, Matagalpa 1021A, Residencial Zacatenco, Gustavo A. Madero, Ciudad de México 07369, Mexico; 7 Departamento de Física y Química Teórica, Facultad de Química, Universidad Nacional Autónoma de México, Circuito Escolar sin Número, Ciudad Universitaria, Alcaldía Coyoacán, Ciudad de México 04510, Mexico

**Keywords:** finite element analysis, dental biomechanics, zirconia Y-TZP, lithium disilicate, stress distribution, full-coverage crown, mandibular molar, stress shielding

## Abstract

The mechanical compatibility between dental restorative materials and the natural tooth structure is a relevant factor for long-term clinical performance. Although zirconia (yttria-stabilized tetragonal zirconia polycrystal, Y-TZP) and lithium disilicate are widely used for full-coverage crowns, their biomechanical interaction with the underlying dentin and pulp under functional loading remains insufficiently characterized. This study reports a comparative finite element analysis (FEA) of a mandibular first molar under vertical (200 N, axial) and oblique (200 N, 30°) loading, evaluating three configurations: an intact healthy tooth, a zirconia Y-TZP full-coverage crown, and a lithium disilicate full-coverage crown. The three-dimensional geometry was obtained from a cone-beam computed tomography (CBCT) study of a caries-free mandibular first molar, previously described and verified by the present group, and was analyzed in ANSYS Workbench (Static Structural). Von Mises equivalent stress, maximum principal stress and total deformation were obtained for enamel or restoration, dentin, and pulp in each configuration. Zirconia produced the highest stress concentrations in the coronal restoration (88.4 MPa vertical; 174.5 MPa oblique), exceeding the healthy enamel baseline by 57.6% and 89.7%, respectively. Both restorative materials reduced dentin stress relative to the healthy tooth, consistent with the stress-shielding effect driven by elastic-modulus mismatch. Under oblique loading, the maximum principal stress in healthy enamel reached 61.7 MPa, approaching or exceeding the upper bound of the reported tensile strength range (~10–40 MPa) and identifying oblique loading as the more demanding of the two conditions analyzed. Within the limitations of the present finite element model, lithium disilicate demonstrated a more favorable stress distribution, with dentin stress values closer to the intact-tooth baseline. The model does not include a luting cement layer, a periodontal ligament, the dentin–enamel junction, anisotropic tissue behavior or cyclic loading, and no experimental validation was performed; the results are therefore presented as a controlled numerical comparison between three configurations under the specific conditions simulated, and not as direct clinical selection criteria.

## 1. Introduction

The selection of restorative materials for dental crowns involves a complex interplay between aesthetic requirements, mechanical resistance, and biomechanical compatibility with the remaining tooth structure. Zirconia (yttria-stabilized tetragonal zirconia polycrystal, Y-TZP) and lithium disilicate glass-ceramics have become the dominant materials for full-coverage restorations because of their favorable aesthetic outcomes and high flexural strength values of 900–1200 MPa and 360–500 MPa, respectively [1,2]. However, the clinical assumption that greater material hardness translates into improved restoration performance overlooks a fundamental biomechanical principle: elastic-modulus compatibility between the restorative material and the natural tooth tissues that it replaces.

Natural enamel has an elastic modulus of approximately 70 GPa, whereas dentin exhibits a substantially lower value of approximately 18 GPa [3]. Zirconia, with a modulus of 200 GPa, is nearly three times stiffer than enamel, and lithium disilicate, at 95–105 GPa, also exceeds the natural tissue values. This mismatch has direct implications for how occlusal forces are transmitted through the restored tooth to the underlying dentin and periodontal structures. Stiffer restorations concentrate stress within the coronal material while shielding the underlying dentin—a phenomenon known as stress shielding—which may alter the physiological stress environment of dental tissues and ultimately affect long-term tissue remodeling and clinical longevity [4].

Finite element analysis (FEA) has become a well-established tool for evaluating stress distributions in dental structures under functional loading. Previous studies have examined individual materials or specific loading scenarios, but systematic comparisons that include a healthy-tooth baseline under both axial and oblique loading remain scarce. Oblique loading is of particular clinical relevance, since functional masticatory movements generate lateral force components that have been identified as the primary mechanism of cusp fracture and restoration failure [5,6].

Recent finite element studies have addressed the biomechanical behavior of zirconia and lithium disilicate restorations from several complementary perspectives. Dal Piva et al. [7] compared monolithic full-crown restorations fabricated from materials of differing stiffness and related the resulting stress fields to the elastic modulus of the crown material. Lin et al. [8] evaluated crown design and material in endodontically treated molars, and Abtahi et al. [9] analyzed endocrown cavity designs in CAD-CAM materials under static vertical load, both reporting that the material–design combination modified the resulting stress concentration. Epifania et al. [10] demonstrated in an implant-supported configuration that stiffer crowns retain a greater proportion of the stress within their own structure while reducing the demand on the supporting components, and Campaner et al. [11] reported an analogous dependence on core stiffness in posterior fixed partial dentures. Nokar et al. [12] extended this observation to implant components and peri-implant bone, and Karn et al. [13] compared lithium disilicate and monolithic zirconia endocrowns on a mandibular molar, showing that the material–design combination modifies the location and not only the magnitude of the peak stress. Sichi et al. [14] reported the same stiffness-driven behavior for zirconia crowns on endodontically treated teeth, and Shrestha et al. [15] showed, using an experimentally validated viscoelastic model, that material compatibility also governs the residual stress state generated during processing.

Despite this body of evidence, three gaps persist. First, most comparative analyses evaluate restorative materials against each other but omit an intact, unrestored tooth as a common reference, so the magnitude of the departure from physiological stress transmission cannot be quantified. Second, a large proportion of these studies apply axial loading only, or report oblique loading without a paired axial condition on the same geometry, which prevents the direct attribution of the observed differences to load direction. Third, the simultaneous reporting of Von Mises equivalent stress, maximum principal stress and total deformation for enamel or restoration, dentin and pulp within a single controlled comparison is uncommon, although these three descriptors are jointly required in order to separate global mechanical demand from tensile fracture risk and from structural compliance. The present study addresses these three points within a single geometry, so that the only parameter modified between models is the coronal material.

The purpose of the present study was to develop and compare three-dimensional FEA models of a mandibular first molar in its healthy state and restored with zirconia Y-TZP and lithium disilicate full-coverage crowns, subjecting them to vertical and oblique (30°) loading in order to quantify stress distributions across enamel or restoration, dentin, and pulp. The null hypothesis of the study was that no difference would be found in the stress behavior between the intact tooth and the two restored configurations under the conditions of the study; the alternative hypothesis was that the elastic-modulus mismatch between restorative materials and natural tooth structure would govern the biomechanical response of the models, with lithium disilicate producing a stress distribution closer to that of the intact tooth than zirconia. The study is conceived as a controlled numerical comparison; accordingly, its outcomes describe the relative biomechanical behavior of the three configurations under the specific conditions simulated and are not intended as direct clinical recommendations.

## 2. Materials and Methods

### 2.1. Geometric Model

**Figure 1:**
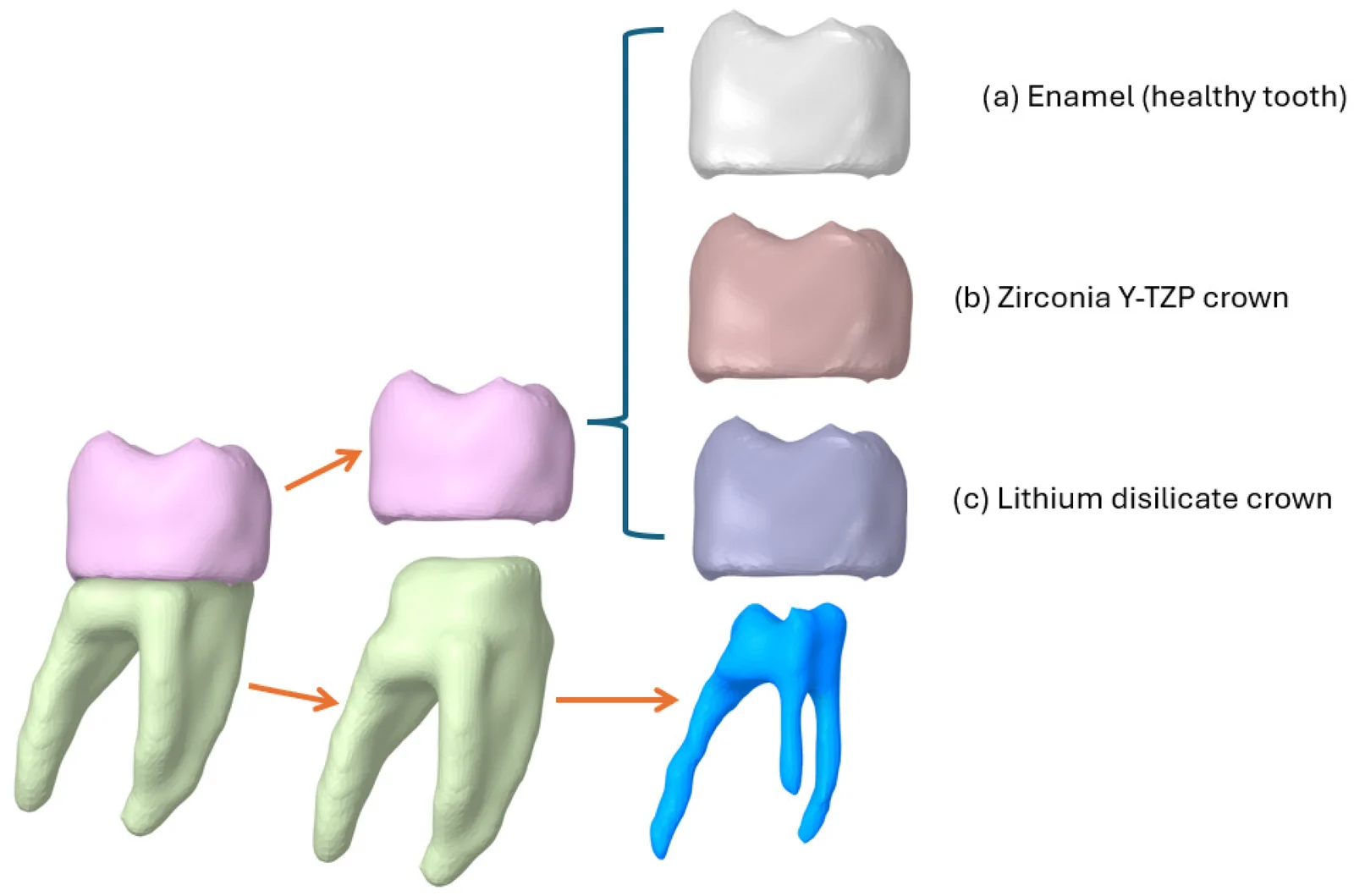
Three-dimensional geometric models of the mandibular first molar showing the exploded view of tissue components and restorative configurations: enamel/restoration layer (top), dentin body (center), and pulp chamber (right): (a) healthy enamel; (b) zirconia Y-TZP full-coverage crown; and (c) lithium disilicate full-coverage crown. Y-TZP, yttria-stabilized tetragonal zirconia polycrystal.

The three-dimensional geometric model of a mandibular first molar was obtained from a cone-beam computed tomography (CBCT) study of a caries-free, unrestored mandibular first molar. The image acquisition parameters, the segmentation protocol, the surface reconstruction procedure and the biofidelity verification of this biomodel have been described in detail in a previous methodological publication by the present research group [16]; the same verified geometry is used here in order to ensure continuity and reproducibility between studies. The model reproduces the anatomical regions of enamel, dentin and pulp chamber, including the cuspal anatomy relevant to occlusal contact distribution. Three configurations were analyzed: (a) intact healthy tooth with all natural tissues; (b) zirconia Y-TZP full-coverage crown replacing the entire enamel volume; and (c) lithium disilicate full-coverage crown replacing the entire enamel volume (Figure 1). In the restored models the enamel volume was replaced by the corresponding restorative material, while the dentin and pulp geometry remained unchanged. This configuration corresponds to a full-coverage crown restoration, in which the enamel is clinically removed and the crown seats directly on the dentin core; the substitution therefore represents the material condition of a complete crown preparation. What this convention does not reproduce is the additional reduction in the dentin core, the change in stump geometry and the finish line created during clinical tooth preparation, nor the difference in wall thickness between restorative materials, points addressed in Section 2.6 and examined in Section 4.5. It also fixes the external geometry, the occlusal contact area and the total volume of the three models to be strictly identical, so that the only parameter modified between models is the elastic modulus of the coronal material. All models were processed and meshed in ANSYS Workbench 2025 R2 (ANSYS Inc., Canonsburg, PA, USA).

### 2.2. Material Properties

All materials were modeled as linear-elastic, homogeneous and isotropic, in agreement with the majority of comparative FEA studies in dental biomechanics [3,17]. This assumption is the prevailing convention in studies with a primary objective to compare restorative materials, as it isolates the effect of the material variable and enables direct comparison with the existing body of FEA literature [18]. The natural tissues nevertheless depart from this idealization in three respects that are relevant to the interpretation of the results. Enamel is orthotropic, with mechanical properties that depend on prism orientation; Munari et al. [19] compared anisotropic and isotropic enamel in a premolar model under axial and oblique loading and reported that the isotropic assumption preserved the location of the stress concentrations while overestimating the tensile stress magnitudes. Dentin exhibits an anisotropy of the order of 7–35% depending on tubule orientation and depth, and its orthotropic modeling reduces displacement and strain magnitudes without reversing the relative stress trends between configurations [20]. The dental pulp is a soft connective tissue that has been represented as a low-modulus elastic solid in finite element studies of the pulp chamber [21], although its actual behavior is viscoelastic and its time-dependent response is not captured by a linear-elastic formulation; because the pulp stresses obtained in all configurations were three to four orders of magnitude below those of the mineralized tissues, this simplification is not expected to influence the coronal comparison, although it precludes any inference regarding pulpal fluid dynamics or proprioceptive response. The isotropic formulation adopted here is therefore expected to affect the absolute values more than the relative ordering between configurations, and this expectation is examined further in Section 4.5. The mechanical properties assigned to each tissue and restorative material are summarized in Table 1. The elastic modulus of dental pulp was set to 0.002 GPa (2 MPa), consistent with its nature as a soft connective tissue, as reported in the literature [22]. Properties of the restorative materials were taken from peer-reviewed primary sources reporting experimentally determined values for zirconia Y-TZP [1,23] and lithium disilicate [2,24].

### 2.3. Finite Element Mesh

**Figure 2:**
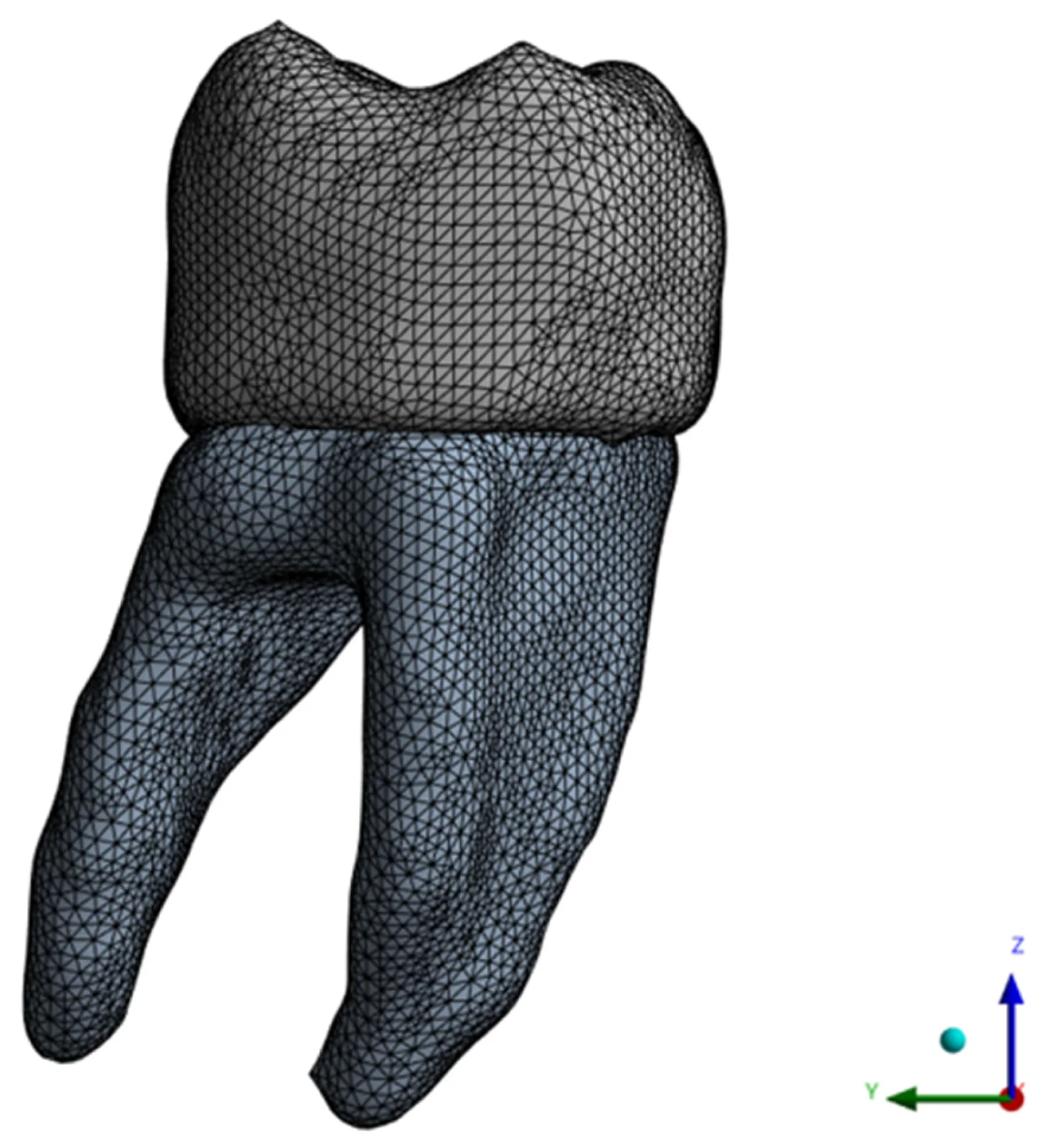
Finite element mesh of the healthy tooth model showing the element density distribution across enamel, dentin, and pulp regions.

All models were discretized using 10-node quadratic tetrahedral elements (SOLID187), with local mesh refinement in the regions of highest stress concentration, namely the occlusal surface, the cervical margin, and the dentin–enamel (or dentin–restoration) interface. The discretization of the reference model comprised 129,005 nodes and 74,907 elements. A mesh-convergence study was performed by progressively refining the element size and comparing the peak Von Mises stress between successive meshes; convergence was defined as a variation below 5% between consecutive refinements. The peak Von Mises stress varied by less than 3% between the final two mesh densities, confirming that the reported discretization (129,005 nodes; 74,907 elements) provided a converged solution (Figure 2). The resulting stress magnitudes for the healthy-tooth model were consistent with the ranges reported in previous finite element studies of intact molars under comparable occlusal loading [5,6]. This agreement constitutes a qualitative consistency check against published magnitudes and not a validation of the model; the scope and limits of this comparison are addressed in Section 4.6.

### 2.4. Boundary Conditions and Loading

A fixed support was applied to the entire root surface, constraining all translational and rotational degrees of freedom at the root boundary. This approach is consistent with previous FEA dental studies focusing on coronal stress distribution, in which the periodontal ligament (PDL) is not explicitly modeled [5,25]. Constraining the root directly increases the overall rigidity of the assembly and modifies the strains transmitted to the supporting structures; Özcan et al. [26] showed that the degree of anatomical complexity represented in a dental finite element model, including the way the supporting structures are described, changes the computed magnitudes without necessarily altering the ordering between configurations. The influence of this boundary condition is greatest on root, cervical and alveolar stress and on tooth mobility, and comparatively smaller on the coronal stress fields evaluated here under static loading. Because the identical constraint was applied to the three models, the comparison between them is not affected by this boundary condition, whereas the absolute values obtained in the cervical and radicular regions are model-specific and should not be interpreted as physiological. This limitation is addressed further in Section 2.6 and Section 4.5.

**Figure 3:**
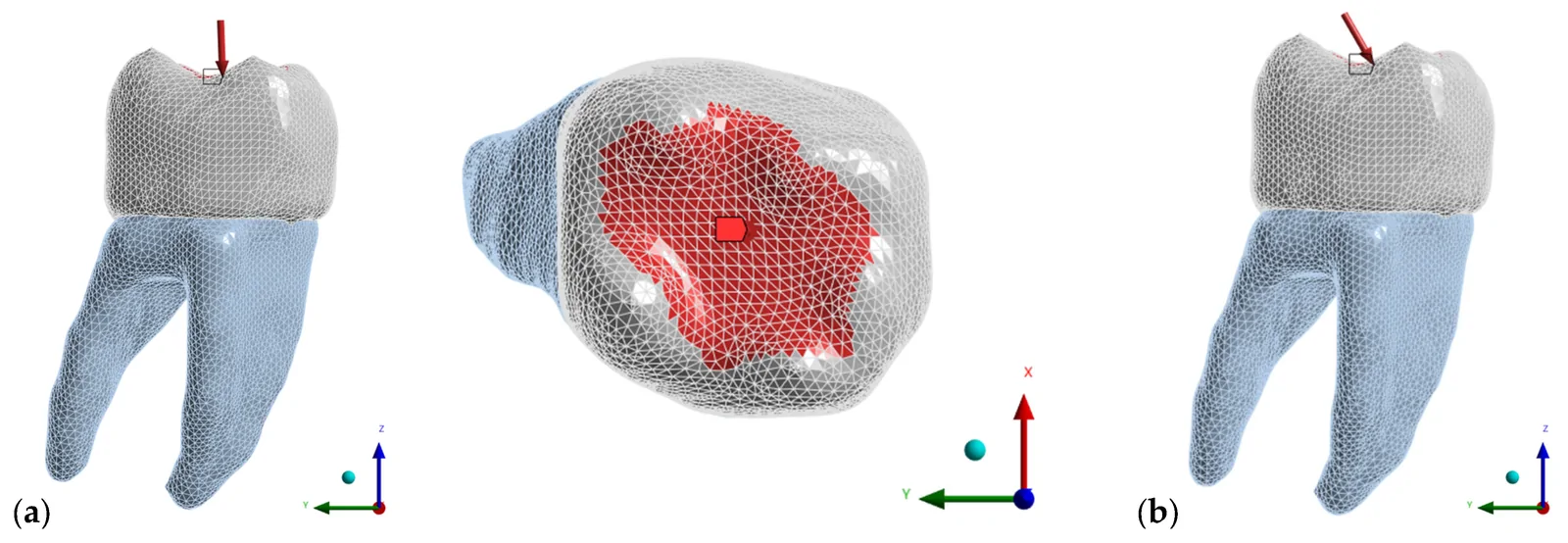
Schematic representation of the loading conditions: (**a**) vertical loading at 200 N along the tooth axis; (**b**) oblique loading at 30° with decomposed axial and horizontal pressure components.

Two loading conditions were applied as uniformly distributed pressure over the occlusal contact area (49.73 mm^2^), representing the functional contact region (Figure 3):

**Vertical loading:** A compressive pressure of 4.02 MPa (equivalent to 200 N distributed over the contact area) was applied along the longitudinal axis of the tooth (*Z*-axis).

**Oblique loading (30°):** A resultant force of 200 N was decomposed into axial (Z) and horizontal (Y) pressure components, P_Z_ = 3.48 MPa (173 N/49.73 mm^2^) and P_Y_ = 2.01 MPa (100 N/49.73 mm^2^), simulating lateral excursive movements during mastication.

### 2.5. Contact Conditions and Analysis Type

All interfaces between adjacent structures (enamel–dentin, restoration–dentin, and dentin–pulp) were defined as bonded contacts, assuming perfect adhesion with no sliding or separation. A linear static-structural analysis was performed in ANSYS Workbench 2025 R2 (ANSYS Inc., Canonsburg, PA, USA). Two output variables were extracted for each tissue in each model: (i) the Von Mises equivalent stress (σ_VM_), used as a global indicator of mechanical demand, and (ii) the maximum principal stress (σ1), used to assess tensile risk in brittle materials and tissues.

### 2.6. Model Simplifications and Their Rationale

The present model incorporates four simplifications that are common in comparative dental FEA but that condition the interpretation of the results. They are stated explicitly here, and their expected influence on the reported magnitudes is examined in Section 4.5.

(i) Absence of a luting cement layer: Clinical crowns are always cemented, and the luting interface, typically 30–100 μm thick, modifies the transmission of stress between the restoration and the underlying dentin. Guler et al. [27] showed that in a crowned molar, the choice of luting agent alone changes the peak Von Mises stress by more than a factor of two. A cement layer was not included here because its thickness and properties would have to be assumed rather than measured for the present configuration, and because the introduction of a third material with an independent elastic modulus would confound the single variable under study. The three models are therefore compared under identical, idealized interfacial conditions.

(ii) Absence of the periodontal ligament: A fixed support was applied at the root surface instead of an explicit PDL, as described in Section 2.4. This choice keeps the boundary condition identical across the three models but removes the shock-absorbing function of the ligament and increases the overall rigidity of the assembly.

(iii) Substitution of the entire enamel volume by the restoration: The configuration corresponds to a full-coverage crown preparation, in which the enamel is removed and the crown seats on the dentin core. What it does not reproduce is the additional geometric reduction in the stump, the finish line, and the difference in preparation depth between materials: zirconia permits thinner walls and therefore a more conservative reduction in tooth structure than lithium disilicate, an advantage that the present fixed-envelope geometry does not represent.

(iv) Absence of the dentin–enamel junction and linear-elastic, homogeneous, isotropic material behavior: The dentin–enamel junction is a graded, multilevel interface with a mechanical function to arrest cracks propagating between the two mineralized tissues [28]; it is represented here as a bonded contact of zero thickness. The consequences of the isotropic and linear-elastic assumptions for enamel, dentin and pulp are described in Section 2.2.

## 3. Results

### 3.1. Von Mises Stress Distribution Under Vertical Loading

**Figure 4:**
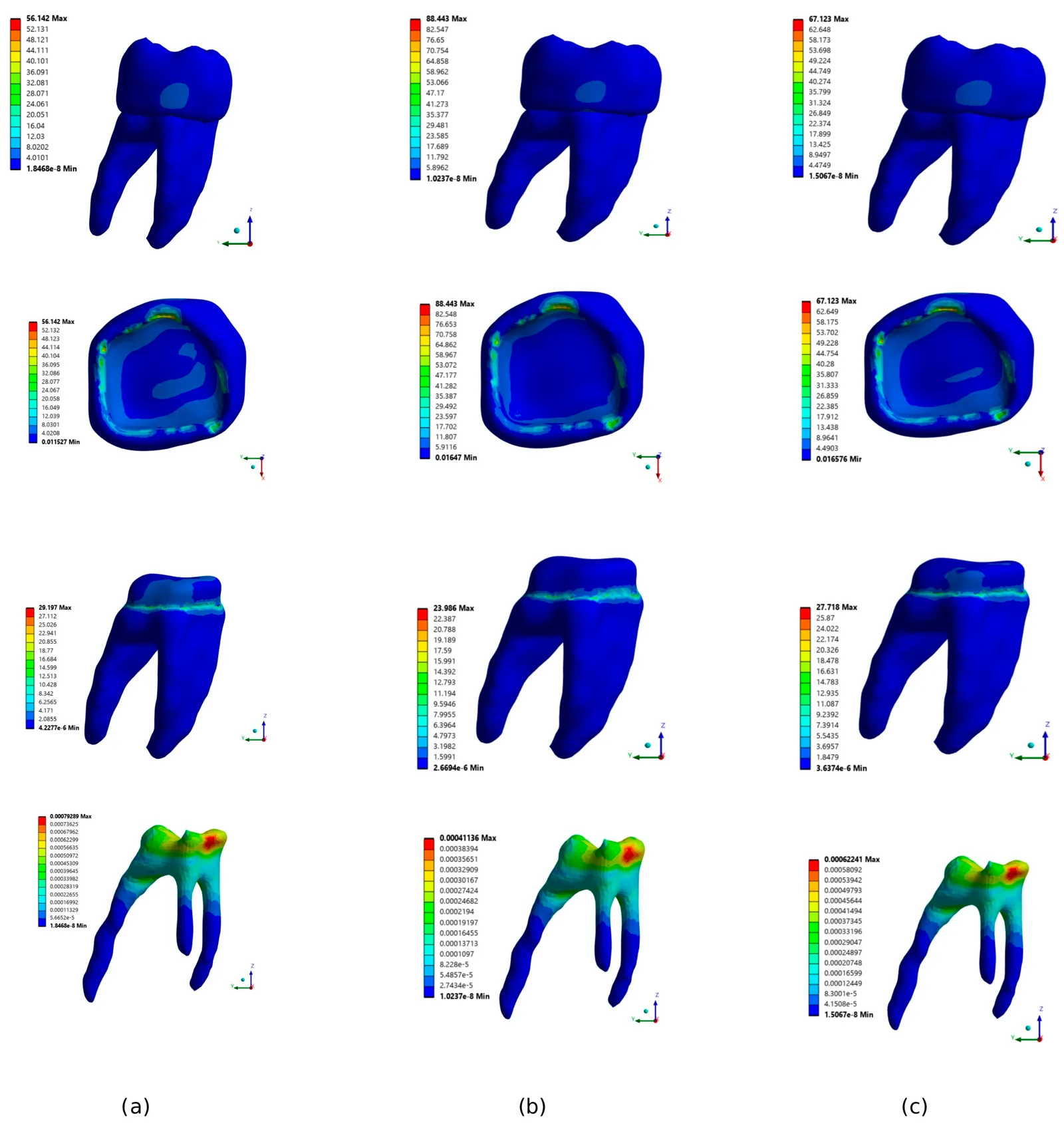
Von Mises equivalent stress distribution under vertical loading (200 N) in (**a**) the healthy molar, (**b**) the zirconia Y-TZP full-coverage crown configuration, and (**c**) the lithium disilicate full-coverage crown configuration. Each column shows, from top to bottom, the external molar view, the occlusal cross-section, the isolated dentin body, and the isolated pulp chamber. Each panel retains its own colour scale; peak values are reported in Table 2.

Under vertical loading (200 N axial), the maximum Von Mises stress in all models was located at the enamel or restoration surface (Table 2). Zirconia produced the highest coronal stress at 88.4 MPa (Figure 4b), representing a 57.6% increase over the healthy enamel baseline of 56.1 MPa (Figure 4a). Lithium disilicate yielded an intermediate value of 67.1 MPa (Figure 4c), corresponding to a 19.6% increase relative to the healthy tooth. Conversely, in the dentin layer, both restorative materials exhibited lower stress than the healthy tooth (zirconia: 24.0 MPa; lithium disilicate: 27.7 MPa; healthy: 29.2 MPa), demonstrating a stress-shielding effect. Pulp stress values were negligible in all configurations (<0.001 MPa), in line with its low elastic modulus and protected anatomical location.

### 3.2. Maximum Principal Stress Under Vertical Loading

**Figure 5:**
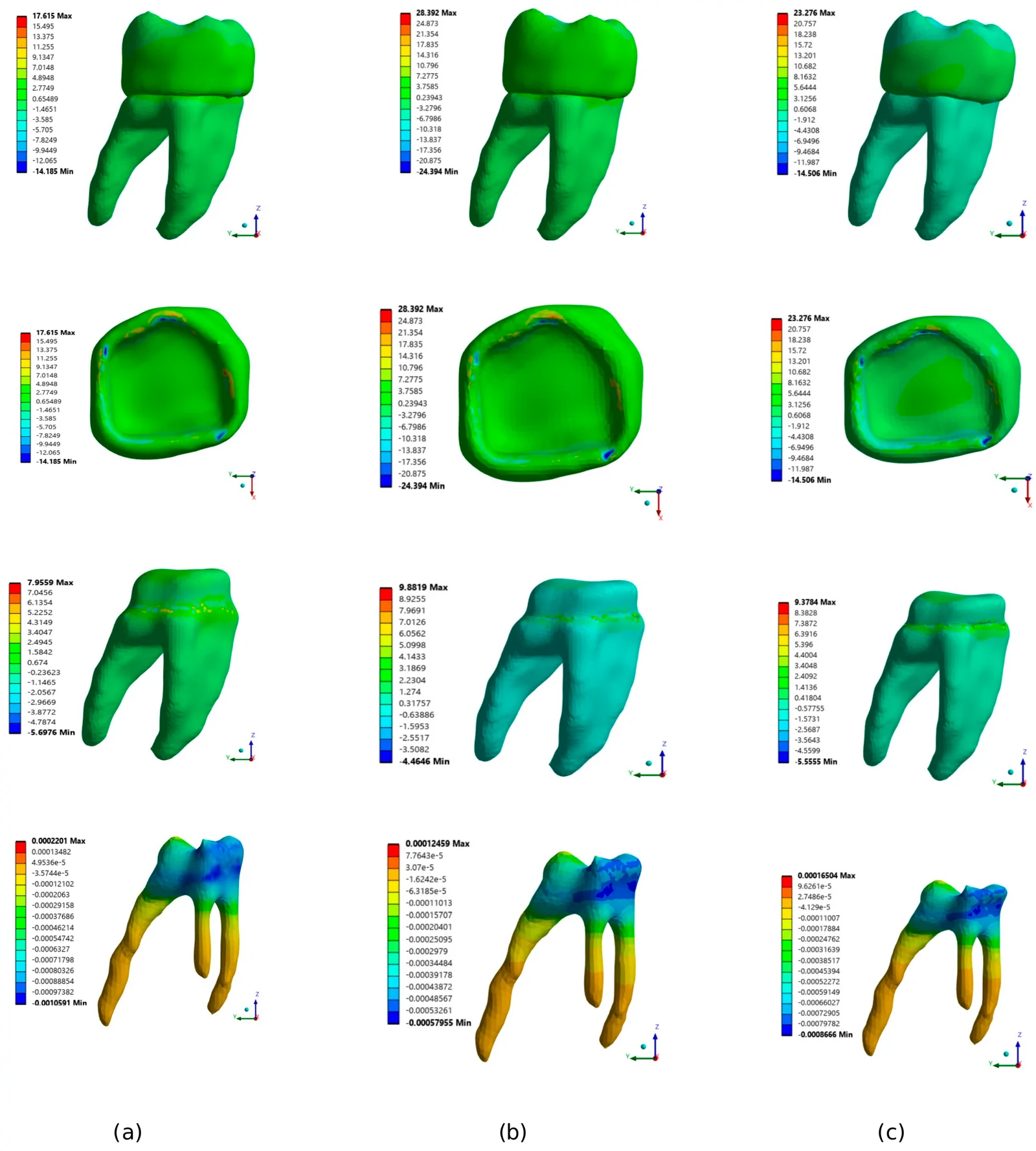
Maximum principal stress distribution under vertical loading (200 N) in (**a**) the healthy molar, (**b**) the zirconia Y-TZP full-coverage crown configuration, and (**c**) the lithium disilicate full-coverage crown configuration. Each column shows, from top to bottom, the external molar view, the occlusal cross-section, the isolated dentin body, and the isolated pulp chamber. Each panel retains its own colour scale; peak values are reported in Section 3.2.

Maximum principal (tensile) stress values under vertical loading remained below the lower bound of the flexural strength reported for each restorative material, and, in the healthy-tooth model, within the reported tensile strength range of enamel (~10–40 MPa). Healthy enamel reached 17.6 MPa (Figure 5a), while the zirconia and lithium disilicate restorations exhibited values of 28.4 MPa (Figure 5b) and 23.3 MPa (Figure 5c), respectively. Dentin tensile stresses remained well below the reported tensile strength of dentin (~52 MPa) in all configurations.

### 3.3. Total Deformation Under Vertical Loading

**Figure 6:**
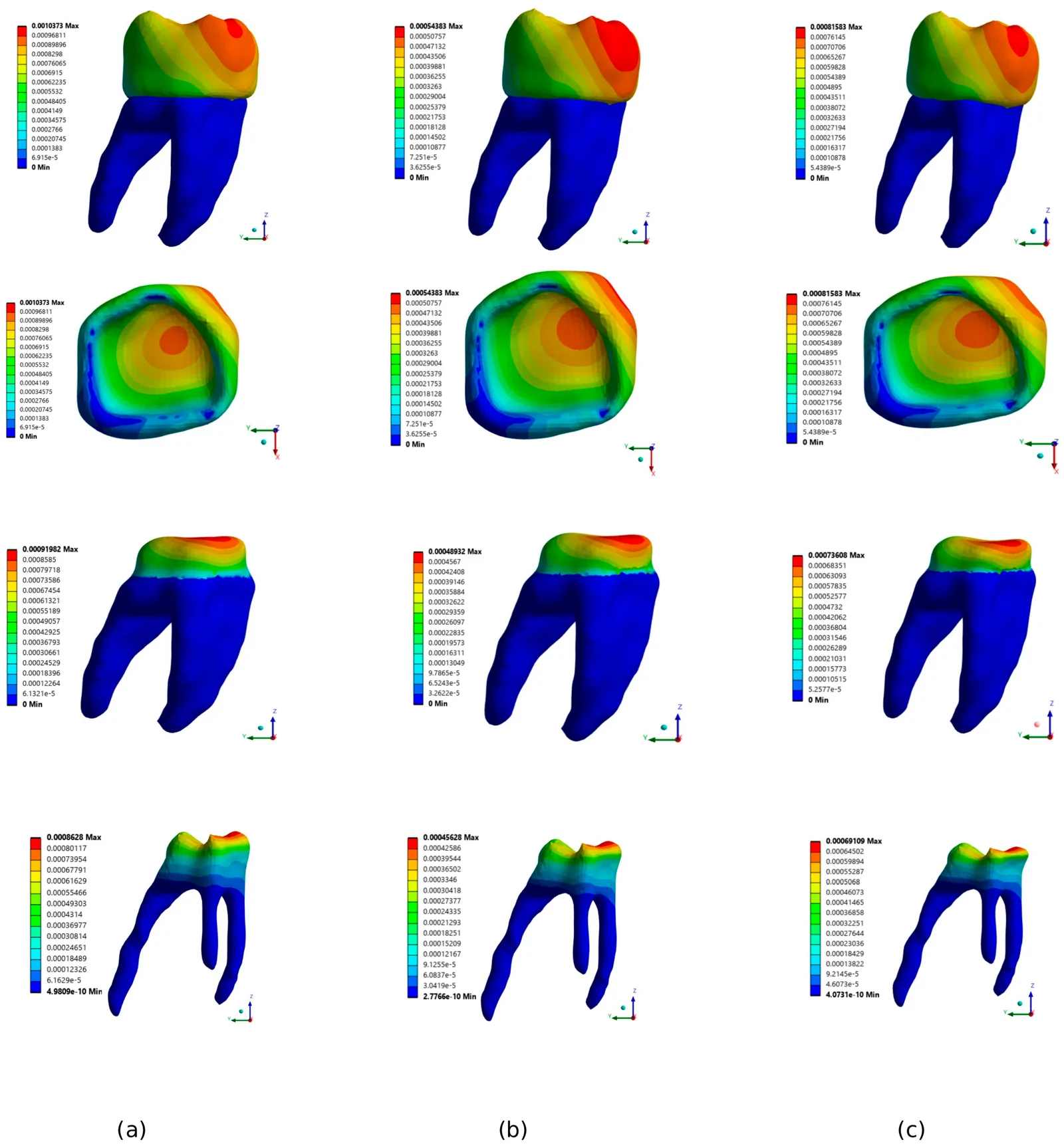
Total deformation distribution under vertical loading (200 N) in (**a**) the healthy molar, (**b**) the zirconia Y-TZP full-coverage crown configuration, and (**c**) the lithium disilicate full-coverage crown configuration. Each column shows, from top to bottom, the external molar view, the occlusal cross-section, the isolated dentin body, and the isolated pulp chamber. Each panel retains its own colour scale; peak values are reported in Section 3.3.

Maximum total deformation under vertical loading was highest in the healthy tooth (1.037 × 10^−3^ mm at the enamel surface; Figure 6a), followed by lithium disilicate (8.158 × 10^−4^ mm; Figure 6c) and zirconia (5.438 × 10^−4^ mm; Figure 6b). The inverse relationship between elastic modulus and deformation is consistent with theoretical predictions: the stiffest material (zirconia) deformed the least, while the natural tooth—with its lower-modulus enamel layer—exhibited the largest displacement.

### 3.4. Von Mises Stress Distribution Under Oblique Loading

**Figure 7:**
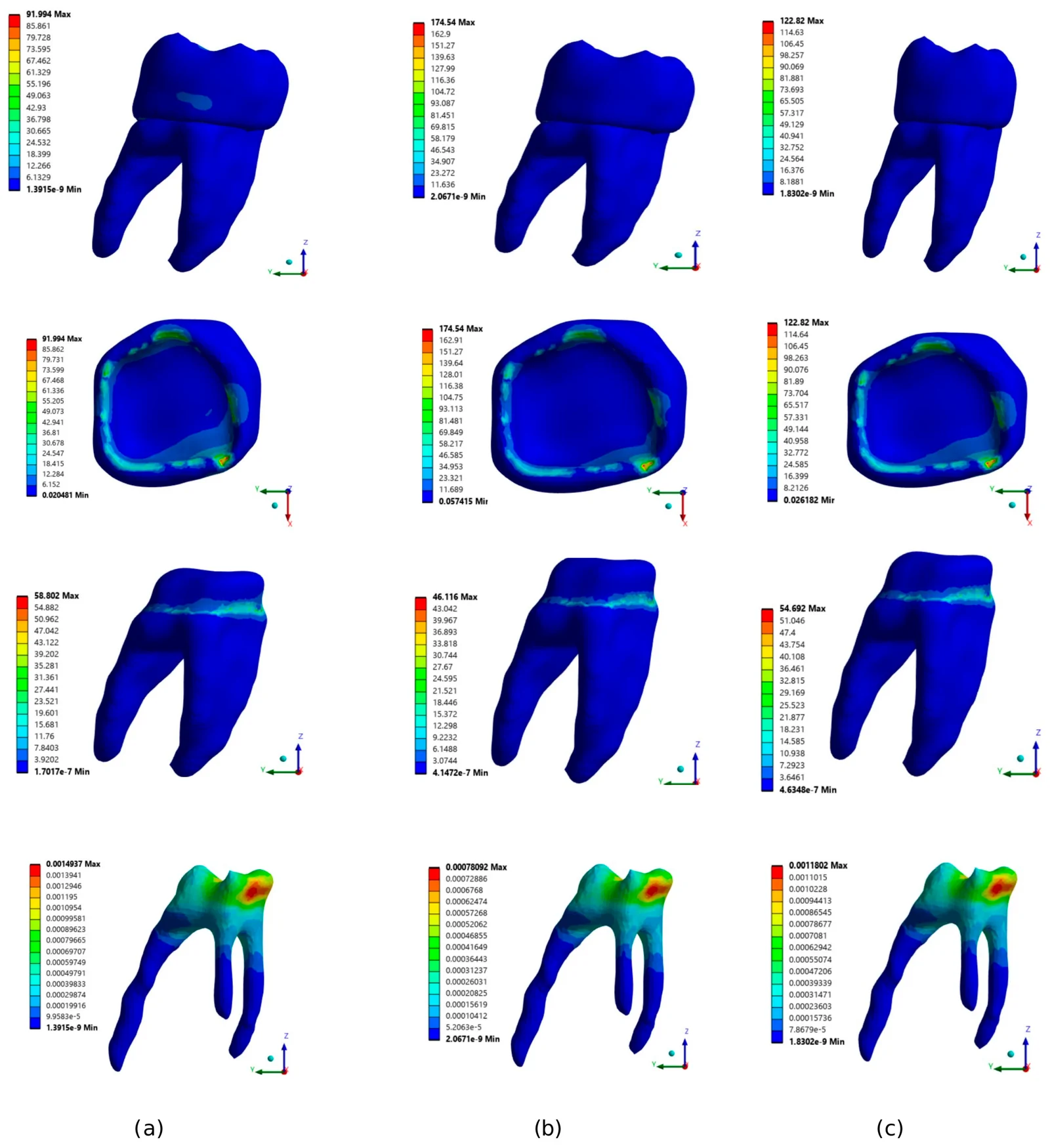
Von Mises equivalent stress distribution under oblique loading (200 N, 30°) in (**a**) the healthy molar, (**b**) the zirconia Y-TZP full-coverage crown configuration, and (**c**) the lithium disilicate full-coverage crown configuration. Each column shows, from top to bottom, the external molar view, the occlusal cross-section, the isolated dentin body, and the isolated pulp chamber. Each panel retains its own colour scale; peak values are reported in Table 3.

Oblique loading (30°, 200 N) produced substantially higher stress magnitudes across all models and tissues compared with vertical loading (Table 3). Zirconia exhibited the highest coronal stress at 174.5 MPa (Figure 7b), an 89.7% increase over the healthy baseline of 92.0 MPa (Figure 7a). Lithium disilicate reached 122.8 MPa (Figure 7c), which is a 33.5% increase. The stress-shielding pattern in dentin was preserved under oblique conditions: zirconia (46.1 MPa) and lithium disilicate (54.7 MPa) both remained below the healthy dentin value (58.8 MPa). The difference between vertical and oblique loading was most pronounced in zirconia, in which the coronal stress nearly doubled (+97%), highlighting the sensitivity of high-modulus restorations to load direction.

### 3.5. Maximum Principal Stress Under Oblique Loading

**Figure 8:**
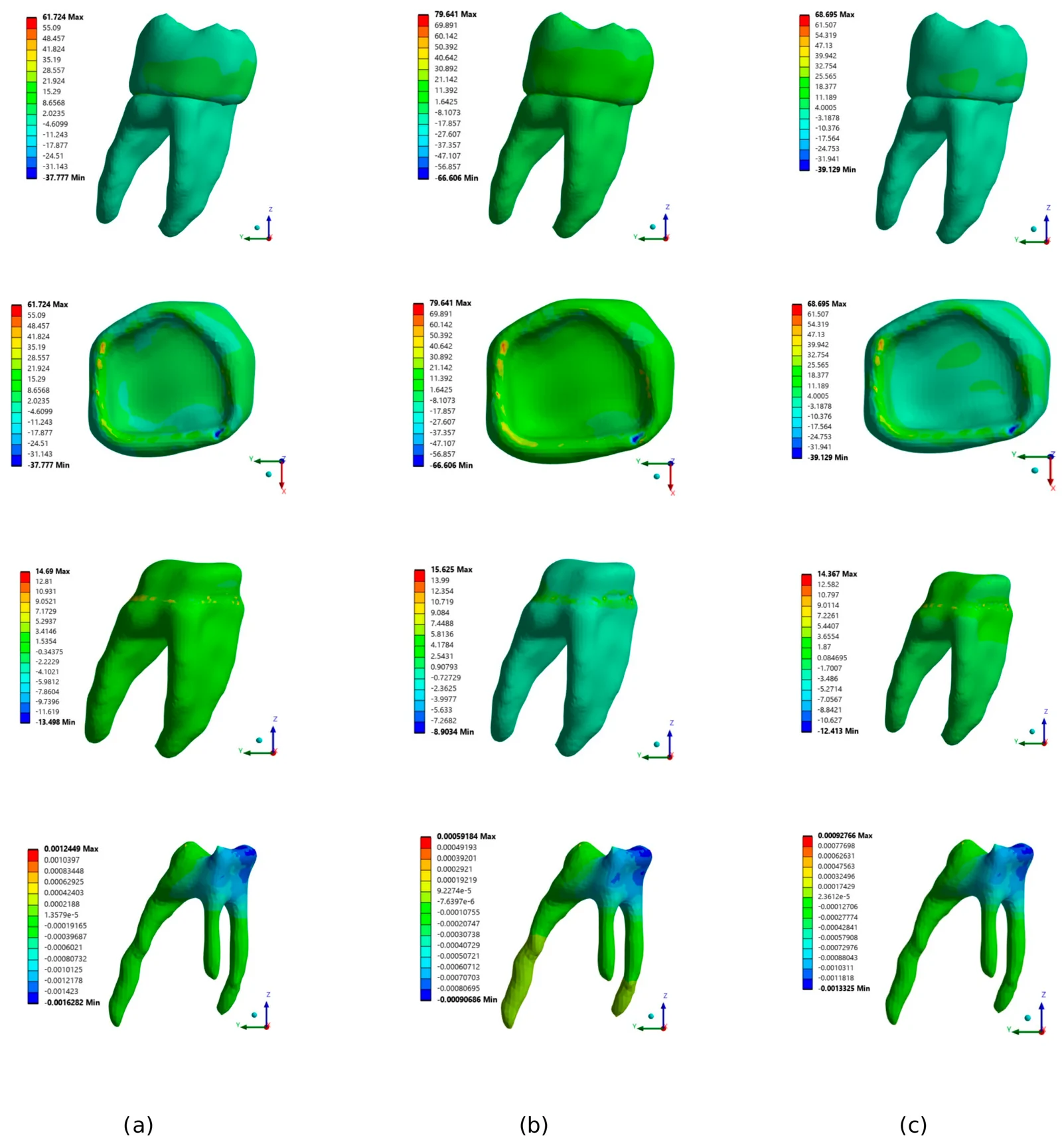
Maximum principal stress distribution under oblique loading (200 N, 30°) in (**a**) the healthy molar, (**b**) the zirconia Y-TZP full-coverage crown configuration, and (**c**) the lithium disilicate full-coverage crown configuration. Each column shows, from top to bottom, the external molar view, the occlusal cross-section, the isolated dentin body, and the isolated pulp chamber. Each panel retains its own colour scale; peak values are reported in Table 4.

Under oblique loading, the maximum principal stress in healthy enamel rose to 61.7 MPa (Figure 8a), approaching or exceeding the upper bound of the reported enamel tensile strength range (~10–40 MPa). The corresponding values at the restoration surface were 79.6 MPa for zirconia (Figure 8b) and 68.7 MPa for lithium disilicate (Figure 8c) (Table 4); these are referred to the flexural strength of each ceramic in Section 4.4. Within the limitations of the present model, and given that the isotropic formulation overestimates tensile magnitudes (Section 4.5), these values should be read as upper-bound estimates rather than as fracture predictions. Dentin tensile stresses remained well below the reported tensile strength of dentin (~52 MPa) in all scenarios.

### 3.6. Total Deformation Under Oblique Loading

**Figure 9:**
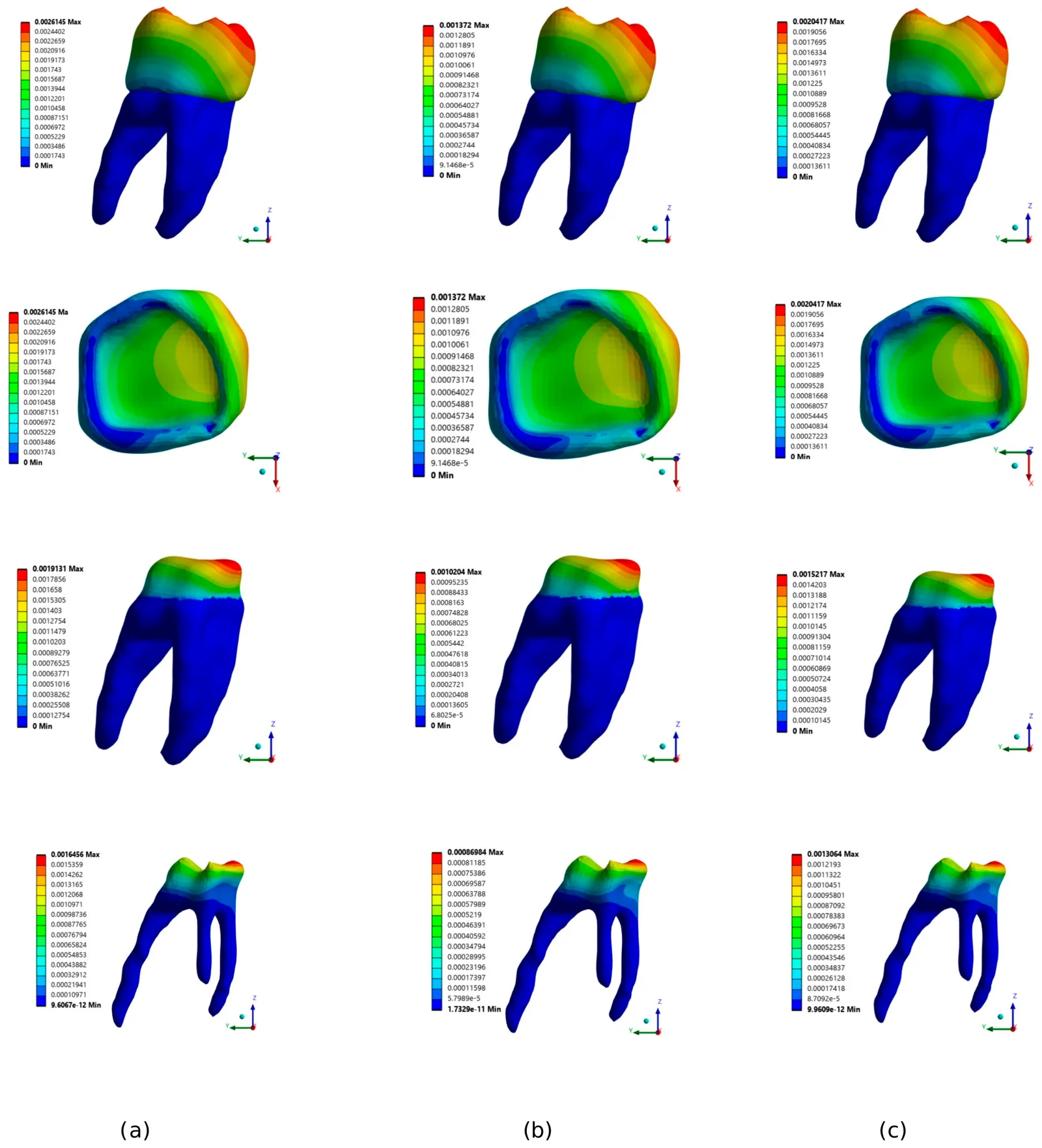
Total deformation distribution under oblique loading (200 N, 30°) in (**a**) the healthy molar, (**b**) the zirconia Y-TZP full-coverage crown configuration, and (**c**) the lithium disilicate full-coverage crown configuration. Each column shows, from top to bottom, the external molar view, the occlusal cross-section, the isolated dentin body, and the isolated pulp chamber. Each panel retains its own colour scale; peak values are reported in Section 3.6.

Under oblique loading, deformation magnitudes increased approximately 2.5-fold relative to vertical loading in all models. The healthy tooth showed the greatest displacement (2.614 × 10^−3^ mm; Figure 9a), followed by lithium disilicate (2.04 × 10^−3^ mm; Figure 9c) and zirconia (1.37 × 10^−3^ mm; Figure 9b). The relative ordering of deformation magnitudes was preserved under both loading conditions, reflecting the dominant role of the elastic modulus of the coronal material.

## 4. Discussion

### 4.1. Elastic-Modulus Mismatch as the Primary Biomechanical Determinant

The present results indicate that, within the conditions simulated, the elastic modulus of the coronal material rather than its flexural strength was the variable that governed the stress distribution across the three configurations. Zirconia, with an elastic modulus of 200 GPa, nearly three times that of natural enamel, concentrated substantially greater stress at the coronal surface while reducing dentin stress through stress shielding. This behavior agrees with the fundamental mechanics of composite structures, in which stiffer components attract a greater proportion of the load under displacement-controlled conditions [18], and with the stiffness dependence reported in comparable dental models [7,10,11,12]. It should be emphasized that this observation derives from a comparison in which the elastic modulus was the only parameter modified between models. The model was not designed to isolate the contribution of mechanical resistance, since no failure criterion or fracture-mechanics formulation was included; the present data therefore support the influence of stiffness within this controlled comparison, and do not establish a general hierarchy between stiffness and strength as determinants of clinical performance.

A restoration that concentrates stress at its own surface while shielding the dentin may therefore behave differently from the intact tooth in ways that are not evident from strength data alone. Should the restoration eventually debond, or should microcracks initiate at the restoration–dentin interface, the previously shielded dentin would be exposed to the full occlusal stress [29]. This sequence is a mechanistic hypothesis consistent with the stress fields obtained here, and not an outcome demonstrated by a static linear-elastic analysis; its verification would require fatigue testing and clinical follow-up.

### 4.2. Comparison with Previous Finite Element Studies

The trends obtained here are consistent with those reported in comparable models. Dal Piva et al. [7], analyzing monolithic full crowns fabricated from materials of differing stiffness under axial load, related the stress transmitted to the substrate to the elastic modulus of the crown; the direction of that relationship corresponds to the 57.6% coronal increase and the 17.8% dentin reduction obtained here for zirconia under vertical loading. Karn et al. [13], comparing lithium disilicate and monolithic zirconia endocrowns on a mandibular molar, reported that the combination of restorative material and margin design modified the resulting stress concentration, which is consistent with the interfacial concentration described in Section 3. Epifania et al. [10] reported for implant-supported crowns that a stiffer crown retains a greater share of the load within its own structure while unloading the underlying components; this reflects the same load-partitioning principle that produces the dentin stress shielding quantified here, although in that study the underlying substrate is titanium and bone rather than dentin, so the parallel is mechanical and not biological. Campaner et al. [11] and Nokar et al. [12] observed an equivalent dependence on core and crown stiffness in fixed partial dentures and in implant components, respectively. The consistency of this ordering across different geometries and substrates suggests that the effect reported here is driven by the elastic-modulus contrast and is unlikely to be an artifact of the present geometry.

The comparison is considerably less direct for the absolute values, and this is informative in itself. Guler et al. [27] reported peak Von Mises stresses of 478.09 MPa and 214.62 MPa in a crowned primary molar under 330 N vertical and oblique load with an explicit 30 μm cement layer. Scaling by the load ratio alone does not reconcile those values with the 88.4 MPa and 174.5 MPa obtained here; the difference is attributable to the combination of a different tooth and crown material, a different occlusal contact area, and above all to the presence of the cement interface, which introduces a compliant interlayer and an additional stress concentrator at the crown margin. This contrast quantifies the sensitivity of absolute stress magnitudes to the modeling assumptions declared in Section 2.6 and supports the restriction of the present conclusions to the comparison between the three configurations analyzed under identical conditions.

With respect to load direction, Munari et al. [19] reported for a premolar model that non-axial loading raises tensile stress in the cervical region, and Özcan et al. [26] showed that model complexity, in particular the representation of the supporting structures and of mandibular kinematics, modifies absolute values without reversing the ordering between configurations. The 64–97% increase in coronal Von Mises stress obtained here on passing from axial to 30° oblique loading is consistent in direction with those reports and supports the identification of oblique loading as the more demanding condition. The magnitude of the increase is not directly comparable across studies, since it depends on the tooth analyzed, the load magnitude and inclination, and the representation of the supporting structures.

### 4.3. Stress Shielding: Interpretation and Scope

The paradoxical reduction in dentin stress observed with both restorative materials (zirconia: −17.8% vertical, −21.6% oblique; lithium disilicate: −5.1% vertical, −7.0% oblique) requires careful clinical interpretation. Although a reduction in dentin stress may appear favorable, prolonged stress shielding has been associated with atrophic changes in biological tissues, analogously to peri-implant bone loss observed under fully osseointegrated implants with high-modulus stems [30]. Although the remodeling capacity of dentin is more limited than that of bone, the alteration of its physiological stress environment warrants consideration in long-term outcome studies.

From this perspective, lithium disilicate produced dentin stress values closer to the healthy baseline (−5.1% versus −17.8% for zirconia under vertical loading). Within the limitations of the present finite element model, lithium disilicate therefore demonstrated a more favorable stress distribution with respect to the intact tooth. Whether this numerical proximity translates into a clinical advantage cannot be established from a static linear-elastic analysis, and the analogy with peri-implant bone remodeling should be read as a mechanistic parallel rather than as evidence of an equivalent biological response in dentin, the remodeling capacity of which is considerably more limited. The clinical preference for lithium disilicate in posterior restorations reported in the prosthodontic literature [2] is therefore consistent with, but not demonstrated by, the present numerical comparison.

### 4.4. Oblique Loading as the More Demanding Loading Condition

The substantial increase in stress magnitudes under oblique loading (a 64–97% rise in coronal Von Mises stress depending on the model) emphasizes the importance of including non-axial loading conditions in FEA dental studies. The maximum principal stress in healthy enamel under oblique loading (61.7 MPa) approaches or exceeds the upper bound of the reported enamel tensile strength range (~10–40 MPa), providing a mechanistic explanation for the clinical observation that dental fractures occur predominantly during lateral excursive movements rather than during maximal intercuspation [31].

For zirconia restorations, the coronal stress under oblique loading (174.5 MPa) represents about 19% of the lower-bound flexural strength (900 MPa); nevertheless, flexural strength values obtained from three-point bending tests do not translate directly to in situ multiaxial stress states and may therefore misrepresent the actual fracture risk. The restoration–dentin interface concentrated stress under oblique loading in both restored models and is accordingly identified as the region of highest mechanical demand within the model. In the present analysis, this interface is a bonded contact of zero thickness, whereas the corresponding clinical interface comprises a luting cement layer and a hybrid layer that degrades over time as a documented failure mechanism [27,29,32]. The present analysis therefore identifies the location of the concentration but cannot predict the failure mode, since neither the cement layer nor a fracture-mechanics criterion was included.

### 4.5. Influence of the Model Simplifications on the Results

The four simplifications declared in Section 2.6 do not affect the present results equally. Their expected influence is examined here in order to delimit which of the reported quantities may be regarded as robust and which are model-specific.

The omission of the luting cement is the simplification with the largest effect on absolute magnitudes. A cement layer with an elastic modulus of the order of 4–8 GPa interposed between a 95–200 GPa restoration and 18 GPa dentin introduces a compliant interlayer that redistributes the load, generates its own stress concentration at the crown margin and alters the gradient across the restoration–dentin interface. The comparison with Guler et al. [27] in Section 4.2 illustrates the magnitude involved. The consequence for the present study is that the interfacial values reported in Section 3 correspond to an idealized, perfectly bonded interface and represent a best case; the introduction of a cement layer would be expected to raise the local stress at the margin and to reduce the degree of dentin stress shielding, because part of the modulus mismatch would be accommodated within the cement rather than transferred directly to the dentin.

The omission of the periodontal ligament increases the overall rigidity of the model. Its effect is concentrated in the root and in the cervical third, where the fixed support imposes a boundary condition that does not exist physiologically; the coronal stress fields evaluated here under static occlusal load are considerably less sensitive to this boundary. Because the three models share the identical constraint and the comparison between them is unaffected, the cervical and radicular values should not be interpreted as physiological, and the model cannot be used to evaluate tooth mobility, load damping or stress transfer to the alveolar bone.

The substitution of the entire enamel volume by the restoration corresponds to the material condition of a full-coverage crown, but does not reproduce three aspects of a clinical preparation: the additional reduction in the dentin core, its change in geometry toward a tapered stump with a defined finish line, and the difference in wall thickness that a clinician would give to zirconia versus lithium disilicate. This last point has a direction: a thinner zirconia crown over a larger residual dentin volume would be expected to reduce the degree of dentin shielding reported here. The present values for the zirconia model therefore likely overestimate the modulus effect relative to a clinically optimized preparation, and the comparison should be read as an evaluation of the material variable under a fixed geometry, and not as a comparison between clinical restorative protocols.

The absence of the dentin–enamel junction and the isotropic, linear-elastic formulation affect chiefly the tensile field and the crack-related interpretation. The dentin–enamel junction is the structure that, in the intact tooth, arrests cracks travelling from enamel into dentin [28]; representing it as a bonded contact of zero thickness removes this toughening mechanism, so the healthy-tooth model does not reproduce the crack-arresting behavior of the natural interface, and the comparison of the healthy configuration against the restored ones is, in this specific respect, conservative. The isotropic assumption, following Munari et al. [19], preserves the location of the concentrations while overestimating tensile magnitudes; consequently, the comparison of the maximum principal stress against the reported tensile strength of enamel in Section 3.5 should be treated as an upper-bound estimate rather than as a fracture prediction.

### 4.6. Validation and Predictive Capability

The numerical behavior of the model was verified through a mesh-convergence study, as described in Section 2.3, and its outputs were compared with magnitudes reported in the FEA literature, as set out in Section 4.2. No experimental validation was performed: neither strain-gauge measurement, nor digital image correlation, nor in vitro fracture testing of the same geometry was available for the three configurations analyzed. The absolute stress values reported here have therefore not been shown to reproduce experimental measurements, and the predictive capability of the model with respect to fracture load or clinical survival is unverified.

What the model does support is the comparison between the three configurations. All of them share the same geometry, the same mesh strategy and element type, the same boundary conditions, the same contact definitions, and the same applied load, so that the differences reported are attributable to the coronal material alone. Experimental validation against strain measurements on extracted molars, and subsequently against fatigue and fracture testing, is the necessary next step before any of these observations can be transferred to clinical decision-making. This constitutes the principal direction of the ongoing work of the present group.

### 4.7. Limitations

Several limitations should be acknowledged, in addition to the four simplifications declared in Section 2.6 and examined in Section 4.5. First, all materials were modeled as linear-elastic, homogeneous and isotropic, which does not capture the anisotropic behavior of enamel and dentin or the viscoelastic behavior of the pulp; future work should incorporate orthotropic material definitions in order to evaluate the extent to which these assumptions modify the tensile field. Second, the periodontal ligament was not explicitly modeled and a fixed support at the root surface was applied instead, which increases the rigidity of the assembly and removes the damping function of the ligament. Third, a single load magnitude of 200 N was applied in only two directions. This value lies within the reported range of maximum bite force in the molar region and was selected in order to permit comparison with published FEA studies that use magnitudes of the same order; because the analysis is linear-elastic, stress scales proportionally with the applied load, so the relative differences between configurations are independent of the magnitude chosen, whereas any threshold-based interpretation, such as the comparison against the tensile strength of enamel, is specific to 200 N. Clinical mastication involves cyclic loading of variable magnitude applied at multiple, migrating contact points, none of which is reproduced by two static load cases. Fourth, a single crown geometry derived from one CBCT study was analyzed; anatomical variation between patients and variation in preparation design may influence the stress distribution, and the present results should not be extrapolated to other tooth types or preparation geometries. Fifth, bond integrity was assumed to be perfect at all interfaces through bonded contacts, whereas in clinical practice adhesive and luting layers possess their own mechanical properties and degrade over time. Sixth, the analysis was performed under static loading conditions, whereas mastication is a cyclic, dynamic process; fatigue behavior was not modeled because the stress–life (S–N) curves required for the restorative materials under the present configuration were not available. Finally, no experimental validation of the model was performed, as discussed in Section 4.6. The evaluation of cyclic fatigue, of the explicit luting interface and of long-term durability, together with experimental validation, therefore, remain the principal directions for future work.

## 5. Conclusions

The present finite element analysis of a mandibular first molar under vertical and oblique loading, comparing a healthy tooth with full-coverage zirconia Y-TZP and lithium disilicate restorations, supports the following observations. All of them apply within the limitations of the model declared in Section 2.6 and examined in Section 4.5 and Section 4.6, and refer strictly to the comparison between the three configurations under the conditions simulated:

1. Within this model, elastic-modulus mismatch was the dominant variable governing the biomechanical response: zirconia, with the highest modulus (200 GPa), generated the greatest coronal stress concentrations, 57.6% and 89.7% above the healthy baseline, under vertical and oblique loading, respectively. The analysis did not include a failure criterion and therefore does not establish a general hierarchy between elastic modulus and mechanical resistance.
2. Oblique loading was the more demanding of the two conditions analyzed: under 30° lateral loading, the maximum principal stress in enamel or restoration approached or exceeded the reported tensile strength range of enamel in all models. This identifies lateral loading as the critical condition within the simulation and is consistent with, although it does not by itself demonstrate, the clinical predominance of fracture during excursive movements.
3. Lithium disilicate demonstrated a more favorable stress distribution within the limitations of the present finite element model, producing dentin stress values closer to those of the intact tooth than zirconia under both loading conditions.

These results quantify the influence of elastic-modulus compatibility on the stress distribution of a restored mandibular molar under two static load cases. They constitute a controlled numerical comparison between three configurations and do not, on their own, establish clinical material-selection criteria; their translation into clinical recommendations requires experimental validation and clinical evidence. Future work should incorporate an explicit cement layer, the periodontal ligament, orthotropic material definitions, a graded representation of the dentin–enamel junction, variable and cyclic loading, and fracture-mechanics criteria, together with experimental validation of the resulting model.

## Figures and Tables

**Table 1:** Mechanical properties of tissues and restorative materials.

Material/Tissue	E (GPa)	ν	ρ (g/cm^3^)	Reference
Enamel	70.0	0.30	2.80	[3]
Dentin	18.3	0.30	2.00	[3]
Pulp	0.002	0.45	1.00	[3]
Zirconia (Y-TZP)	200.0	0.30	6.08	[1,23]
Lithium disilicate	105.0	0.23	2.50	[2,24]

Abbreviations: E (elastic modulus); ν (Poisson’s ratio); ρ (density); Y-TZP (yttria-stabilized tetragonal zirconia polycrystal).

**Table 2:** Maximum Von Mises stress by tissue under vertical loading (200 N).

Tissue	Healthy (MPa)	Zirconia (MPa)	Li-Disilicate (MPa)	Δ vs. Healthy
Enamel/Restoration	56.1	88.4	67.1	+57.6/+19.6%
Dentin	29.2	24.0	27.7	−17.8/−5.1%
Pulp	0.0008	0.0004	0.0006	—

**Table 3:** Maximum Von Mises stress by tissue under oblique loading (200 N, 30°).

Tissue	Healthy (MPa)	Zirconia (MPa)	Li-Disilicate (MPa)	Δ vs. Healthy
Enamel/Restoration	92.0	174.5	122.8	+89.7/+33.5%
Dentin	58.8	46.1	54.7	−21.6/−7.0%
Pulp	0.0015	0.0008	0.0012	—

**Table 4:** Maximum principal (tensile) stress by tissue and loading condition (MPa).

Condition/Tissue	Healthy	Zirconia	Li-Disilicate	Tensile Strength *
Vertical, Enamel/Rest.	17.6	28.4	23.3	~10–40 (enamel) †
Vertical, Dentin	8.0	9.9	9.4	~52 (dentin)
Oblique, Enamel/Rest.	61.7	79.6	68.7	~10–40 (enamel) †
Oblique, Dentin	14.7	15.6	14.4	~52 (dentin)

* Reference tensile strength values from the literature: enamel ~10–40 MPa; dentin ~52 MPa. † The enamel tensile strength range applies to the healthy-tooth model only. The zirconia and lithium disilicate values are referred to the flexural strength reported for each ceramic (900–1200 MPa and 360–500 MPa, respectively [1,2]), as discussed in Section 4.4.

## Data Availability

The data supporting the findings of this study are available from the corresponding author upon reasonable request.
